# Challenges and barriers to musculoskeletal injury research in sub-Saharan Africa: a modified Delphi study

**DOI:** 10.1302/2633-1462.78.BJO-2025-0409.R1

**Published:** 2026-08-14

**Authors:** National Institute for Health and Care Research Global Health Research Group on Global Injury

**Affiliations:** 1 Oxford Trauma and Emergency Care, Nuffield Department of Orthopaedics, Rheumatology & Musculoskeletal Sciences, University of Oxford, Oxford, UK; 1 Orthopaedic Research Unit, University of Cape Town, Cape Town, South Africa; 2 Malawi University of Science and Technology, Thyolo, Malawi; 3 Kamuzu University of Health Sciences, Blantyre, Malawi; 4 Muhimbili University of Health and Allied Sciences, Dar es Salaam, Tanzania; 5 Countess of Chester Hospital NHS Foundation Trust, Chester, UK; 6 AO Alliance Foundation, Chur, Switzerland; 7 University of Oxford, Oxford, UK; 8 Queen Elizabeth Central Hospital, Blantyre, Malawi; 9 Malawi Liverpool Wellcome Research Programme, Blantyre, Malawi; 10 Liverpool School of Tropical Medicine, Liverpool, UK; 11 Lilongwe Institute of Orthopaedics and Neurosurgery, Lilongwe, Malawi; 12 South African Medical Research Council, Cape Town, South Africa; 13 University of Cape Town, Cape Town, South Africa; 14 London School of Hygiene & Tropical Medicine, London, UK; 15 Stellenbosch University, Cape Town, South Africa; 16 Ministry of Health and Social Welfare, Dodoma, Tanzania; 17 University of Liverpool, Liverpool, UK; 18 University of Bristol, Bristol, UK; 19 University of California San Francisco, San Francisco, California, USA; 20 University of Dar es Salaam, Dar es Salaam, Tanzania

**Keywords:** Modified Delphi, Musculoskeletal, Injury, Trauma, Research, Low-and-middle income countries, musculoskeletal injury, healthcare professionals, morbidity, clinicians, Orthopaedic Trauma, general orthopaedic surgeons, physiotherapists, Delphi methodology, strain, clinical trials

## Abstract

**Aims:**

Musculoskeletal (MSK) injuries are a major cause of morbidity and mortality worldwide. However, research into MSK injuries is hampered by significant challenges and barriers, particularly in low-and middle-income countries (LMICs). Our aim was to identify the most important challenges and barriers to MSK injury care research across Malawi, South Africa, and Tanzania.

**Methods:**

We conducted a three-stage modified Delphi study. In Stage 1, healthcare professionals and researchers involved in MSK injury care listed challenges and barriers to conducting MSK injury research, which were consolidated in 25 themes. In Stage 2, participants rated each theme on a five-point Likert scale. In Stage 3, to drive consensus, participants re-rated the themes after viewing group and country-specific feedback from the previous stage. Challenges and barriers were ranked by mean score. Consensus was defined as ≥ 75% of respondents rating a theme as 4 or 5 (‘important’ and ‘most important’ on the Likert Scale).

**Results:**

A total of 396 participants completed Stage 1, 424 completed Stage 2, and 434 completed Stage 3. Across all participants, consensus (≥ 75%) was reached on nine key challenges and barriers to MSK injury research, including: lack of funding, high clinical workloads, limited training and mentorship, poor-quality data collection practices, healthcare facility resource constraints, patient loss to follow-up, limited research opportunities outside academic institutions, poverty and financial barriers, and healthcare delays. At the country level, the challenges of lack of funding, high clinical workload, and inconsistent data collection practices consistently achieved consensus in Malawi, South Africa, and Tanzania.

**Conclusion:**

MSK injuries impose a substantial burden in sub-Saharan Africa, yet research remains limited by funding shortages, high clinical workloads, and inconsistent data collection practices. These challenges, consistently identified across Malawi, South Africa, and Tanzania, highlight the need for targeted investment and regional collaboration to strengthen research capacity and improve patient outcomes.

Cite this article: *Bone Jt Open* 2026;7(8):1076–1083.

## Introduction

Musculoskeletal (MSK) injuries are a major contributor to morbidity and disability worldwide, with a disproportionate burden in low- and middle-income countries (LMICs), particularly across sub-Saharan Africa (SSA).^1-3^ In many LMICs poor road infrastructure, rapid urbanization, and poorly established trauma and injury care systems exacerbate both the frequency and severity of injuries. Additionally, safe and affordable surgical and trauma care remain inaccessible to many. Beyond the immediate clinical consequence, MSK injuries lead to long-term disabilities, reduce quality of life, and have a significant economic and social cost through lost productivity, household financial strain, and impaired community participation.^4-7^ At a national level, injuries lower productivity, strain healthcare systems, and reduce gross domestic product (GDP) growth. Despite the scale of the problem, MSK injury research remains limited in SSA and LMICs, restricting progress in injury prevention, care improvement, and policy development.^8^

Many hospitals in LMICs lack electronic health records and reliable trauma registries, making it difficult to generate or analyze data on injury numbers, management, and outcomes.^9^ Research efforts are further constrained by lack of institutional support for clinician-researchers, inconsistent ethical review processes, and limited opportunities for multidisciplinary collaboration among doctors, nurses, physiotherapists, and allied healthcare workers.^10^ Furthermore, MSK injury research is rarely prioritized by international funders, with most resources directed towards communicable diseases such as HIV, tuberculosis, and malaria, despite the higher burden of trauma and injury. When conducted, such research is often driven by external priorities rather than local needs.^11,12^ While these challenges have been described previously, they are not specific to MSK injury research, and there remains a paucity of literature incorporating the perspectives of frontline stakeholders managing these injuries on a daily basis across LMICs, where the majority of injuries occur.

To understand why MSK injury research remains underdeveloped in this region, we sought to identify the challenges and barriers to conducting such research and to highlight those considered most important, thereby providing a foundation for targeted efforts to strengthen research capacity and ultimately improve patient care. We undertook a consensus-building exercise using a modified Delphi approach, hereafter referred to as the POINT Study (Priorities and Objectives in Orthopaedic Trauma Research) which aimed to define the challenges to conducting MSK injury research across Malawi, South Africa, and Tanzania. The study drew on the expertise of clinicians and researchers directly engaged in MSK healthcare delivery and research in these countries.

## Methods

### Modified Delphi method

This study was conducted through the National Institute for Health and Care Research (NIHR) Global Health Group on Global Injury (GIG), an equitable partnership between collaborative researchers across high-, middle-, and low-income countries with the shared vision of improving the care of the injured through research. We conducted a modified Delphi study (POINT Study) to identify challenges and barriers to conducting MSK research and to assess their relative importance, focusing on Malawi (low-income country), South Africa (upper-middle-income country), and Tanzania (lower-middle-income country) between February and August 2025. These countries were purposively selected to reflect a range of health system contexts and World Bank income classifications within SSA;^13^ while not representative of all SSA countries, this approach enabled exploration of shared and context-specific barriers across diverse settings.

A three-stage electronic survey was administered in English. Participants included healthcare professionals (doctors, nurses, physiotherapists, and other clinicians involved in MSK injury care), as well as researchers working in MSK research. The survey was disseminated through professional networks coordinated by the GIG members in each country and through open calls shared on social media platforms. All participants were invited from the first stage, but new participants could also join at any stage. Although the survey was primarily intended for stakeholders with experience working within MSK injury care or research in our three partner countries, its wide distribution resulted in responses from participants in other countries as well. Eligibility was self-declared and these individuals were included to maximize inclusivity and capture a broad range of relevant perspectives (Supplementary Material).

**Fig. 1:**
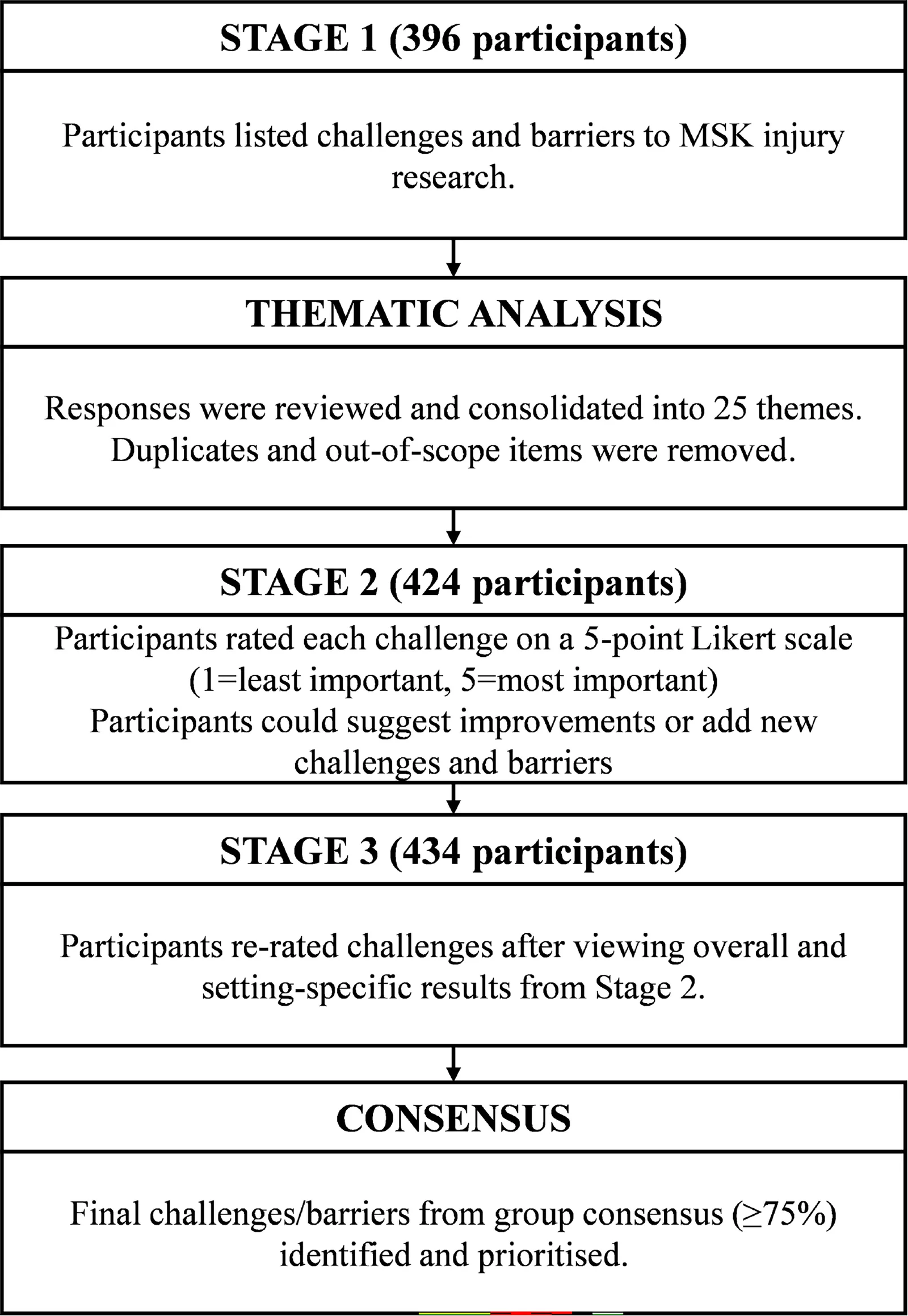
Overview of the modified Delphi process and individual stages. Surveys were disseminated via national networks, country leads, professional societies, and open social media calls. The total number of individuals reached could not be determined. The figure also shows the number of participants in each stage. Some participants completed more than one stage (overlapping), while others were new participants in later stages, consistent with the open recruitment strategy. MSK, musculoskeletal.

The three stages were as follows. Stage 1: an initial scoping survey was distributed to identify a comprehensive list of challenges and barriers to conducting MSK research with the use of a free-text response field. All free-text responses from Stage 1 were reviewed and characterized by the GIG steering committee, consisting of clinicians and MSK researchers, into 25 priority areas (Supplementary Material). This was performed by removing duplicate and non-research-related challenges and barriers, and consolidating the remaining items into thematic categories that encompassed the full range of the issues identified. Stage 2: a second survey was distributed to rate the list of 25 priority areas, on a five-point scale (1 = least important, 5 = most important), based on its importance in hindering research in each country. Participants were encouraged to suggest refinements or add new challenges and barriers not already included after the Stage 1 survey. Stage 3 involved a third survey in which participants re-rated the list of 25 challenges and barriers, with ratings from Stage 2 provided in the form of summary bar graphs to allow participants to reconsider their opinions in the context of responses from their own country, as well as the overall group, after viewing aggregated results from both levels (Figure 1).

The survey was administered electronically through REDCap (Research Electronic Data Capture),^14^ a secure web-based application for online surveys and databases. For Stage 1 and 2, the survey was open for completion for four weeks, and for Stage 3, five weeks. To be included in the data analysis,^15^ respondents were required to answer every question at the stage of the survey they were completing but did not have to complete all three stages.

The study was approved by University of Cape Town Human Research Ethics Committee (HREC REF:212/2024) and participant consent was obtained at the time that the survey was administered.

### Statistical analysis

The results in the Delphi Round 2 and 3 survey were summarized with aggregate ratings to show distribution of responses across the 5-point Likert rating scale (1 = least important, 5 = most important). For each item, we report the mean (SD) scores. For Stage 3, we report the consensus percentages across all survey participants, and separately for each individual partner country (Malawi, South Africa, and Tanzania). Consensus was calculated by taking the proportion of participants who rated it as ‘4’ or ‘5’ (‘important’ or ‘most important’) and defined as ≥ 75% of participants rating a challenge as 4 or 5. Challenges and barriers meeting this threshold were considered to have reached consensus. This threshold is widely used in Delphi methodology, particularly in health research, as it represents substantial agreement while avoiding overly restrictive criteria that may exclude important items.^16-18^ Combining Likert categories 4 and 5 is also consistent with previous Delphi studies where the upper two points of the scale are often grouped to reflect meaningful agreement on importance.^19^

## Results

A total of 396 individuals participated in Stage 1, generating over 1,000 individual challenges and barriers. Eight entries were deemed not relevant and removed from the dataset. Of all challenges and barriers identified, 204 (20%) specifically highlighted the lack of dedicated funding as a major obstacle. The remaining responses were grouped into the relevant 25 consolidated themes. In Stage 2, 424 participants rated the 25 consolidated challenges and barriers. Additional challenges suggested during Stage 2 were reviewed by the steering committee, and either considered not directly relevant to the initial research question, or were found to overlap with one or more of the 25 previously identified challenges barriers. As a result, no new challenges were added to the framework for Stage 3. In Stage 3, 434 participants re-rated the consolidated challenges and barriers in order to drive a consensus on those considered most important (Figure 1).

Across all stages, most participants in Malawi were clinical officers (40%), followed by nurses (17%), whereas they were consultants in South Africa (44%) and Tanzania (23%) followed by residents/registrars (South Africa: 18% and Tanzania: 40%) (Table I). Additional ‘other’ primary professional roles are listed in the Supplementary Material.

**Table I.** Split of primary professional roles across all participating countries and across all three Delphi stages.

Country	Malawi			South Africa			Tanzania			All other countries		
Primary professional role	Stage 1	Stage 2	Stage 3	Stage 1	Stage 2	Stage 3	Stage 1	Stage 2	Stage 3	Stage 1	Stage 2	Stage 3
Clinical Officer	35	59	40	0	0	0	1	1	0	0	0	0
Consultant	7	4	8	66	35	50	50	30	30	9	9	23
Fellow	0	0	0	3	2	2	9	2	3	0	0	3
Intern	0	2	7	1	19	2	2	8	2	0	1	0
Medical Officer	0	0	0	6	5	14	15	12	7	2	1	1
Nurse	13	19	25	5	12	2	18	15	44	0	0	0
Occupational therapist	0	0	0	1	1	0	0	1	0	0	0	0
Physiotherapist	17	11	11	4	2	2	6	17	13	0	0	1
Researcher/Academic	3	6	3	12	8	8	2	2	2	2	3	6
Resident/registrar	7	10	9	25	19	21	41	59	38	0	6	10
Theatre staff	0	1	0	0	0	0	5	6	3	0	0	0
Other	10	15	12	2	8	5	15	8	11	0	1	10
Not specified	0	0	1	0	2	1	2	1	4	0	1	0
Total	92	127	116	125	113	107	166	162	157	13	22	54

Across all three partner countries, as well as other countries represented in the survey, most participants were general orthopaedic surgeons (Malawi: 73%, South Africa: 30%, Tanzania 64% and all other countries: 39%) (Table II).

**Table II.** Split of main orthopaedic specialties across all participating countries and across all three Delphi stages.

Country	Malawi			South Africa			Tanzania			All other countries		
Main orthopaedic speciality	Stage 1	Stage 2	Stage 3	Stage 1	Stage 2	Stage 3	Stage 1	Stage 2	Stage 3	Stage 1	Stage 2	Stage 3
Arthroplasty	1	0	0	7	4	6	8	5	6	0	3	1
Foot & ankle	0	0	1	3	2	2	1	0	0	1	0	0
General	63	91	91	41	31	32	98	111	103	4	9	22
Hand	1	2	1	3	3	4	1	0	0	1	0	0
Limb reconstruction	0	0	0	8	6	8	1	0	1	1	1	3
Oncology	0	1	0	4	1	4	0	0	0	0	1	1
Paediatrics	2	3	3	7	4	3	2	5	3	1	0	3
Shoulder and elbow	1	1	1	9	2	4	0	0	0	0	0	0
Soft-tissue knee	0	0	0	2	3	2	2	1	0	0	0	0
Spines	3	0	1	3	3	6	8	6	6	0	0	2
Trauma	17	21	12	26	25	26	35	24	28	4	6	17
Other	4	8	6	12	29	10	10	10	10	1	2	5
Total	92	127	116	125	113	107	166	162	157	13	22	54

The top ten challenges and barriers are presented in Table III, ranked by overall mean score. Consensus was achieved for nine of the top ten challenges and barriers.

**Table III.** Top ten challenges and barriers (C/B), ranked by the mean score percentages for all participants from Stage 3 and across each partner country (Malawi, South Africa, Tanzania).

Ranking	All participants			Malawi			South Africa			Tanzania		
	**C/B**	**Mean (SD)**	**Consensus, %**	**C/B**	**Mean (SD)**	**Consensus, %**	**C/B**	**Mean (SD)**	**Consensus, %**	**C/B**	**Mean (SD)**	**Consensus, %**
1	Funding	4.54 (0.8)	89	Clinical workload	4.65 (0.72)	90	Clinical workload	4.73 (0.58)	95	Funding	4.55 (0.80)	89
2	Clinical workload	4.46 (0.9)	85	Training and mentorship	4.64 (0.89)	91	Data collection practices	4.45 (0.80)	86	Training and mentorship	4.31 (0.94)	83
3	Training and mentorship	4.28 (0.9)	81	Funding	4.63 (0.72)	91	Funding	4.42 (0.89)	88	Poverty/financial barriers	4.26 (1.08)	78
4	Data collection practices	4.23 (1)	80	Healthcare facility resource constraints	4.59 (0.72)	93	Healthcare delays	4.05 (0.97)	78	Clinical workload	4.20 (1.19)	77
5	Healthcare facility resource constraints	4.19 (1)	79	Data collection practices	4.40 (0.84)	84	Loss to follow-up	4.04 (0.75)	84	Publication costs	4.18 (1.03)	75
6	Loss to follow-up	4.14 (1)	78	Healthcare delays	4.39 (1.02)	81	Healthcare facility resource constraints	3.94 (0.99)	79	Incentives and recognition	4.14 (1.09)	76
7	Limited research opportunities outside academic institutions	4.13 (0.9)	75	Incentives and recognition	4.38 (0.93)	83	Limited research opportunities outside academic institutions	3.90 (0.81)	73	Limited research opportunities outside academic institutions	4.13 (0.99)	74
8	Poverty/financial barriers	4.13 (1.1)	75	Limited research opportunities outside academic institutions	4.38 (0.91)	82	Training and mentorship	3.88 (0.93)	70	Loss to follow-up	4.13 (1.12)	77
9	Healthcare delays	4.09 (1.1)	75	Research infrastructure	4.34 (1.00)	81	Data analysis/statistical support	3.84 (0.97)	69	Healthcare facility resource constraints	4.12 (1.14)	72
10	Research infrastructure	4.07 (1.1)	73	Loss to follow-up	4.28 (1.01)	74	Poverty/financial barriers	3.82 (0.98)	68	Research infrastructure	4.11 (1.14)	73

## Discussion

In this multinational modified Delphi study, we identified key challenges and barriers to conducting MSK research, with a focus on three SSA countries: Malawi, South Africa, and Tanzania. From over 1,000 responses, 25 themes encompassing the challenges and barriers were established and rated by participants in two subsequent stages. Across all participants, the top ten challenges and barriers were the lack of dedicated research funding, high clinical workload, limited access to training and mentorship, inconsistent and poor data collection practices, healthcare facility resource constraints, patient loss to follow-up, limited research opportunities outside academic institutions, poverty and financial barriers limiting patient enrolment, follow-up and participation, healthcare delays, and insufficient research infrastructure. Of these ten challenges and barriers, nine achieved consensus (≥ 75%) across all participants in Stage 3. When disaggregated by country, lack of funding, high clinical workload, and inconsistent data collection practices consistently achieved consensus as the most important challenges and barriers to conducting MSK research in Malawi, South Africa, and Tanzania. These highlight the multifaceted barriers to conducting MSK research injury in LMICs, particularly within SSA. Addressing these barriers is essential to strengthen MSK injury research capacity, to generate contextually relevant data and evidence, and ultimately improve patient outcomes. These challenges highlight the structural and systemic obstacles that must be addressed to build sustainable MSK research capacity in SSA.^2,20,21^ Addressing them will require coordinated solutions, including increased investment, protected research time for clinicians, strengthening of hospital infrastructure, and innovative strategies for patient retention.

Across Malawi, South Africa, and Tanzania, participants underscored interrelated domains affecting research activity and capacity. Funding was the most universal limitation across all participants. Funding should be proportionate to the substantial global health burden MSK injuries impose.^22^ There are few dedicated funding streams for MSK injury research, despite the high burden particularly in LMICs and SSA, and minimal institutional recognition or financial incentives and motivation for clinician-researchers to conduct research. Inadequate funding limits both research infrastructure and research capacity and support, affecting the scope and quality of MSK research studies and leading to challenges in data collection and analysis. Limited funding also constrains research capacity and skills, as opportunities for mentorship, training, and statistical support are scarce, particularly outside academic institutions.

Weak infrastructure and institutional support, including inadequate research facilities, lengthy ethics and approval processes to conduct research, and limited collaboration mechanisms, further hampers MSK research progress. Even when research is initiated, poor data systems and practices (such as inconsistent data collection, limited access to existing data, and lack of data sharing frameworks) compromise the quality and reproducibility.

At the health system level, clinical and health system barriers, notably high clinical workload, healthcare delays, resource shortages, and fragmented referral systems limit the ability of clinicians to participate in research studies or sustain ongoing research efforts. Finally, sociocultural and contextual factors, including poverty, geographical and rural barriers, cultural and linguistic challenges, and high out-of-pocket patient costs reduce participation in studies and contribute to patient loss to follow-up. This influences the feasibility of conducting longitudinal and/or multicentre studies, and compromises data completeness, study valididity, and outcome assessments.

Collectively, these domains reveal that MSK injury research in SSA is shaped by structural inequities in funding and support, alongside local health system and contextual realities. These findings highlight critical areas where targeted interventions, policy changes, and resource allocation could have the greatest impact. Addressing these barriers requires coordinated investment in research infrastructure, workforce development, and capacity building, and context-driven funding priorities that align with local needs rather than external agendas.

The absence of robust MSK injury research infrastructure has tangible consequences for patients and health systems. Without reliable data, the true burden of MSK injury remains unknown, limiting health system planning, workforce allocation, and surgical capacity development. Poor follow-up and incomplete outcome data impede evaluation of patient outcomes and cost-effectiveness, constraining quality improvement and continuing inefficiencies in care delivery. Targeted investment in MSK research, protected research time, and locally led capacity building would enable accurate burden estimation, inform context-appropriate service design, support policy advocacy, and improve continuity and quality of patient care. Crucially, strengthened research systems would also facilitate pragmatic clinical trials of simple, low-cost interventions tailored to local settings, enabling evidence-based improvements in care delivery and patient outcomes. Maintaining the status quo risks continued underinvestment, fragmented services, and avoidable disability in a population already bearing a disproportionate injury burden.

Our findings complement and extend prior consensus studies in trauma and injury research both globally and in LMICs. A global scoping review and Delphi study highlighted substantial gaps in equity, rehabilitation, and prevention indicators, particularly in LMIC contexts by identifying nearly 500 potential performance indicators for MSK trauma systems. Through this study a core set of indicators focusing on access to care, essential hospital resources, and timely management of common fracture emergencies was identified.^23^ This study largely reflected literature from high-income settings and identified limited attention to equity or post-hospital care. Our work builds on this by directly engaging clinicians and researchers from SSA to define regionally relevant challenges and barriers to MSK research. Members of our group have previously highlighted the significant challenges in conducting injury research in LMICs, including resource and infrastructure limitations, data scarcity, limited research capacity, and limited policy and advocacy, all of which hinder research in this field.^24^ A previous study among early-career cardiologists identified similar challenges, including limited research funding, insufficient mentorship, inadequate institutional support, and barriers to publication.^25^ This closely mirrors the challenges we found in the context of MSK injury research in our partner countries, which suggests that the systemic barriers to conducting clinical research in LMICs are shared across specialties, reinforcing the need for broader capacity-building initiatives and infrastructural support.^25^

The strengths of our study lie in the multicountry SSA representation including LMIC diversity and the inclusive, iterative Delphi method with consensus from stakeholders. While this study focused on three countries, future work could expand to a broader range of SSA and other LMIC contexts globally, to capture a more comprehensive regional and global perspective. In addition, mixed-methods approaches, such as qualitative interviews and implementation research, would provide deeper insights into the underlying challenges and barriers, and identify practical strategies to strengthen MSK research systems.

The study also had several limitations. We used a snowballing approach to broaden the reach to, and the diversity of, expert voices. As surveys were disseminated through country research members, professional networks, and open social media calls, the total number of individuals invited could not be determined. Instead, we report the number of participants who responded in each stage. Although all experts identified in the first stage were invited to subsequent rounds, additional participants could contribute at any stage through redistribution of the survey link or open calls via social media. This approach was adopted to maximize inclusivity and capture perspectives from a wider range of stakeholders across SSA. As a result, new participants entering later stages may have different baseline views that were not highlighted in earlier stages. Consequently, we were unable to directly assess how individual participants changed their ratings following feedback. Instead, consensus was evaluated at the group level within each stage. Additionally, the survey was conducted in English, which may have limited participation from stakeholders who were less fluent in English and potentially influenced the breadth of perspectives captured. Finally, participants self-declared their own legibility to participate in the survey. Therefore, no confirmation was sought of their experience of working within our partner countries or other LMICs.

In conclusion, MSK injuries impose a substantial burden in SSA, yet research remains limited by funding shortages, high clinical workloads, and inconsistent data collection practices. These challenges, consistently identified across Malawi, South Africa, and Tanzania, highlight the need for targeted investment and regional collaboration to strengthen research capacity and improve patient outcomes.

**Take home message**

- This study provides one of the first regionally derived, consensus-based assessments of the barriers to musculoskeletal injury research in sub-Saharan Africa.

- By identifying the challenges that matter most to healthcare workers and researchers across Malawi, South Africa, and Tanzania, it offers a clear roadmap for funders, policymakers, and research networks to prioritize investments in research capacity, data systems, and workforce support.

- Addressing these barriers is essential to generating the evidence needed to improve musculoskeletal injury care and outcomes in the region.

## Data Availability

The data that support the findings for this study are available to other researchers from the corresponding author upon reasonable request.
